# Cross-Reactivity of SARS-CoV-2 Nucleocapsid-Binding Antibodies and Its Implication for COVID-19 Serology Tests

**DOI:** 10.3390/v14092041

**Published:** 2022-09-14

**Authors:** Alexandra Rak, Svetlana Donina, Yana Zabrodskaya, Larisa Rudenko, Irina Isakova-Sivak

**Affiliations:** 1 Institute of Experimental Medicine, Saint Petersburg 197022, Russia; 2 Smorodintsev Research Institute of Influenza, Saint Petersburg 197376, Russia; 3 Institute of Biomedical Systems and Biotechnology, Peter the Great Saint Petersburg Polytechnic University, Saint Petersburg 194064, Russia

**Keywords:** COVID-19, SARS-CoV-2, nucleocapsid phosphoprotein, recombinant protein, cross-reactivity, antibody tests

## Abstract

The emergence of the new coronavirus SARS-CoV-2 in late 2019 led to the global pandemic COVID-19, causing a profound socioeconomic crisis. Adequate diagnostic tools need to be developed to control the ongoing spread of infection. Virus-specific humoral immunity in COVID-19 patients and those vaccinated with specific vaccines has been characterized in numerous studies, mainly using Spike protein-based serology tests. However, Spike protein and specifically its receptor-binding domain (RBD) are mutation-prone, suggesting the reduced sensitivity of the validated serology tests in detecting antibodies raised to variants of concern (VOC). The viral nucleocapsid (N) protein is more conserved compared to Spike, but little is known about cross-reactivity of the N-specific antibodies between the ancestral B.1 virus and different VOCs. Here, we generated recombinant N phosphoproteins from different SARS-CoV-2 strains and analyzed the magnitude of N-specific antibodies in COVID-19 convalescent sera using an in-house N-based ELISA test system. We found a strong positive correlation in the magnitude of anti-N (B.1) antibodies and antibodies specific to various VOCs in COVID-19-recovered patients, suggesting that the N-binding antibodies are highly cross-reactive, and the most immunogenic epitopes within this protein are not under selective pressure. Overall, our study suggests that the RBD-based serology tests should be timely updated to reflect the constantly evolving nature of the SARS-CoV-2 Spike protein, whereas the validated N-based test systems can be used for the analysis of sera from COVID-19 patients regardless of the strain that caused the infection.

## 1. Introduction

In December 2019, a new human infection, SARS-CoV-2, was discovered, causing a global pandemic in early 2020 [1]. To date, over 500 million cases of the new coronavirus disease 2019 (COVID-19) including more than 6 million deaths have been registered worldwide, and new strains of the virus appear regularly [2]. To control the spread of the infection and develop effective strategies to protect public health and the world economy from this pathogen, rapid detection and analysis of COVID-19 cases is necessary.

Laboratory testing for SARS-CoV-2 can be performed in three ways: First, detection of viral antigens by rapid antigenic tests; second, direct detection of viral RNA by real-time PCR of pharyngeal or nasopharyngeal swabs; and third, evaluation of the humoral immune response to infection by serological methods, such as ELISA, microneutralization or automated chemiluminescent immunoassay (CLIA), to detect antibodies and determine their intensity of production [3]. Although serologic testing is not well-suited for detecting acute infections, it supports a number of very important applications. Serologic testing at the population level can not only determine the rate of infection in the affected area and track the spread of infection over time, but also assess the spread of COVID-19 in the community or health care settings and identify at-risk groups [4,5].

The humoral immune response in humans to SARS-CoV-2 infection and vaccination with COVID-19 vaccines is well studied, with a tremendous amount of published data on its intensity and duration (reviewed in [6]). It is known that SARS-CoV-2 cell entry is mediated by interactions between the receptor-binding domain of viral spike glycoprotein and ACE2 host cell receptor; therefore, anti-spike antibodies are considered to be neutralizing [7]. In contrast, antibody levels to nucleocapsid (N) phosphoprotein do not correlate with the neutralizing activity of convalescent sera [8]. Nevertheless, the S and N antigens are widely used in serological tests for SARS-CoV-2 infection as well as for monitoring the immunogenicity of various COVID-19 vaccines [9,10].

Noteworthy, the majority of the antibody tests were developed using the antigens of the ancestral SARS-CoV-2 strain, whereas the virus has significantly evolved during its circulation for more than 2.5 years [11]. Therefore, the sensitivity of these tests for detecting antibodies in patients infected with new SARS-CoV-2 variants remains unclear. While antibody tests targeting Spike are more susceptible to reduced sensitivity due to the high mutation rate of this antigen, N-based test systems are expected to be less affected since the N protein is more conserved. To confirm this assumption, we developed an in-house N-based ELISA test system for the detection of IgG antibodies in serum of COVID-19 convalescents and evaluated the cross-reactivity of induced antibodies against N proteins of different SARS-CoV-2 variants.

## 2. Materials and Methods

### 2.1. Study Participants

Thirty-two serum samples were collected from twenty-two COVID-19 convalescents aged 31 to 84 years who participated in the study of humoral and T-cell responses to SARS-CoV-2, which was approved by the Ethics Committee of the Institute of Experimental Medicine (protocol No. 2/20 on 7 April 2020). The disease onset ranged between May 2020 and October 2021, and time post symptoms onset (PSO) was from 1 to 16 months. The demographic characteristics and the severity of the participants are shown in Table S1. All patients signed an informed consent. Control historical serum samples (n = 36) were obtained from archived specimens collected from healthy adults who participated in Phase I trials of H2N2 (NCT01982331) [12] and H5N2 (NCT01719783) LAIVs [13].

### 2.2. Viruses and Proteins

Five SARS-CoV-2 viruses belonging to different lineages were obtained from the Smorodintsev Research Institute of Influenza (Saint Petersburg, Russia):

- HCoV-19/Russia/StPetersburg-3524/2020 (B.1 Lineage, Wuhan);
- HCoV-19/Russia/SPE-RII-27029S/2021 (B.1.351 Lineage, Beta);
- HCoV-19/Japan/TY7-503/2021 (P.1 Lineage, Gamma);
- HCoV-19/Russia/SPE-RII-32759S/2021 (B.1.617.2 Lineage, Delta);
- HCoV-19/Russia/SPE-RII-6243V1/2021 (B.1.1.529 Lineage, Omicron).

In addition, a human seasonal coronavirus OC43 isolated from an infected subject in the 2020/2021 season, was provided by the Smorodintsev Research Institute of Influenza (Saint Petersburg, Russia). The viruses were amplified in Vero-CCL81 cells as previously described [14] and stored in single-used aliquots at −70 °C.

A recombinant protein encompassing the receptor-binding domain (RBD) of the Spike protein of SARS-CoV-2 (B.1 Lineage) was expressed in eucaryotic cells by BIOCAD JSC (Saint Petersburg, Russia) [15].

### 2.3. Generation of Recombinant N Proteins

Purification of total RNA from the virus-containing liquid was carried out using the Biolabmix RNA Isolation Kit (Biolabmix, Novosibirsk, Russia). All genes encoding the N proteins were amplified using BioMaster RT-PCR Premium Kit (Biolabmix, Novosibirsk, Russia) and cloned into multiple cloning site 1 (MCS-1) of the pETDuet-1 expression vector using BamHI and NotI restriction sites. In this plasmid, the gene inserted in MCS-1 yielded a recombinant protein with a poly-histidine tag at the N terminus. The genes were ligated in pETDuet-1 and the recombinant proteins were expressed in *E. coli* BL21 (DE3) cells using a standard protocol [16].

Purification of N proteins was performed at room temperature using the column packed with His-Bind resin charged with Co2+ [17]. The flow rate at all stages of fractionation was 0.5 mL/min. To remove non-specifically bound proteins, wash buffer (20 mM Tris-HCl, 0.5 M NaCl, 60 mM imidazole, pH 7.9) was used. The bound recombinant N proteins were eluted with buffer containing 20 mM Tris-HCl, 250 mM NaCl, 200 mM imidazole, and pH 7.9. The protein detection was performed at a wavelength of 280 nm using Implen™ NanoPhotometer™ N60.

SDS-PAGE of collected fractions was performed using a running gel with a 12.5% acrylamide concentration and a stacking gel containing 5% acrylamide. Analyzed proteins were dissolved in loading buffer (60–100 μg/mL) and denaturated for 5 min at 95 °C, then 15 μL of samples was loaded onto the gel (1–1.5 μg of protein per lane). SDS-PAGE was performed in Mini-PROTEAN Tetra Cell system (BioRad Hercules, CA, USA) and the results were visualized by staining with Coomassie Brilliant Blue G-250. Recombinant proteins from corresponding bands were hydrolyzed with trypsin (Promega, Madison, WI, USA) in the presence of 50 mM ammonium bicarbonate for 18 h at 37 °C according to the manufacturer’s protocol. The obtained peptides were mixed with DHB matrix (Bruker, Bremen, Germany), coated on the GroundSteel target (Bruker, Germany), and air-dried. Mass spectra (each generated on the basis of 5000 laser pulses) were obtained using the UltrafleXtreme MALDI-TOF/TOF mass spectrometer (Bruker, Germany) in positive ion mode. To identify the proteins, the MASCOT search algorithm [18] with simultaneous access to the SWISS-PROT data bank and to the local database, including pre-added sequences, was used. The mass error did not exceed 20 ppm; methionine oxidation and deamidation were indicated as possible peptide modifications.

Western blot analysis of the recombinant N proteins was performed by wet protein transfer to nitrocellulose membranes using the Mini-PROTEAN Tetra Cell chamber, followed by the blockage of non-specific binding 5% skim milk in PBS+0.05% Tween-20 (PBS-T). Then, the membrane was treated with the HisProbe-HRP (Thermo Fisher Scientific, Waltham, MA, USA), and the color was developed by the addition of 0.05% diaminobenzidine in PBS containing 1% hydrogen peroxide.

### 2.4. Assessment of Virus-Specific Antibody in Human Serum Samples

Serum IgG antibody specific to RBD (B.1) or N proteins were measured by ELISA. For this, 96-well high-sorbent plates (Thermo Fisher Scientific, USA) were coated with RBD or purified recombinant N proteins, 100 ng per well in PBS (pH 7.4) overnight at 4 °C. Then, the plates were blocked with 1% BSA in PBS (pH 7.4) for 40 min at 37 °C, washed 3 times with PBS-T, and 2-fold sera dilutions in PBS-T (1:10 to 1:10,240) were added to the wells and incubated for 1 h at 37 °C. Each sample was tested in duplicate. After incubation, the plates were washed 3 times and an HRP-conjugated goat anti-human IgG secondary antibody (Sigma, St. Louis, MO, USA, cat#AP309P) was added to each well and incubated at 37 °C for 1 h. Then, the plates were thoroughly washed and developed with 1-Step™ Ultra TMB-ELISA Substrate Solution (Thermo Fisher Scientific, USA) for 15 min. After stopping the reaction with 1 M H_2_SO_4_, the absorbance at wavelength 450 nm (OD_450_) was measured using xMark Microplate Absorbance Spectrophotometer (Bio-Rad, Hercules, CA, USA). The area under the OD_450_ curve (AUC) values were calculated for each serum sample using the trapezoidal rule and expressed in arbitrary units.

Virus-neutralizing antibody titers were determined using Vero-CCL81 cells as previously described [14]. Briefly, 2-fold dilutions of heat-inactivated sera were prepared in culture medium, and equal volumes of serum dilutions and live SARS-CoV-2 at a dose of 100 TCID50 were mixed. Then, after 1 h of incubation, they were applied to the Vero cells cultured in 96-well plates. After adsorption, the inoculum was removed and the wells were covered with culture media containing corresponding serum dilutions. After 2–3 days of incubation, the media was removed and the SARS-CoV-2 antigen was detected by cell-ELISA using the polyclonal anti-RBD antibody as a primary antibody and anti-rabbit IgG-HRP conjugate as a secondary antibody. The color was developed with 1-Step™ Ultra TMB-ELISA Substrate Solution (Thermo Fisher Scientific, USA) and the signal was read on xMark Microplate Absorbance Spectrophotometer (Bio-Rad). The 50% neutralizing (MN_50_) titers were calculated using a four-parameter non-linear regression analysis as described in [19].

### 2.5. Statistical Analysis

Data were analyzed using the statistical tool of GraphPad Prism 6.0 Software (GraphPad Software, San Diego, CA, USA). Compliance with the normal distribution was checked by the Shapiro-Wilk test. The significance of differences between two independent groups was assessed by the two-sided Mann-Whitney U test. The significance of differences between two matched groups was assessed by the Wilcoxon matched pairs test. Differences between several test groups were analyzed by one-way ANOVA with Tukey’s multiple comparisons test. The Spearman correlation test was used to evaluate the relationships between the measurements. The significance level was set at *p* < 0.05.

## 3. Results

**Figure 1:**
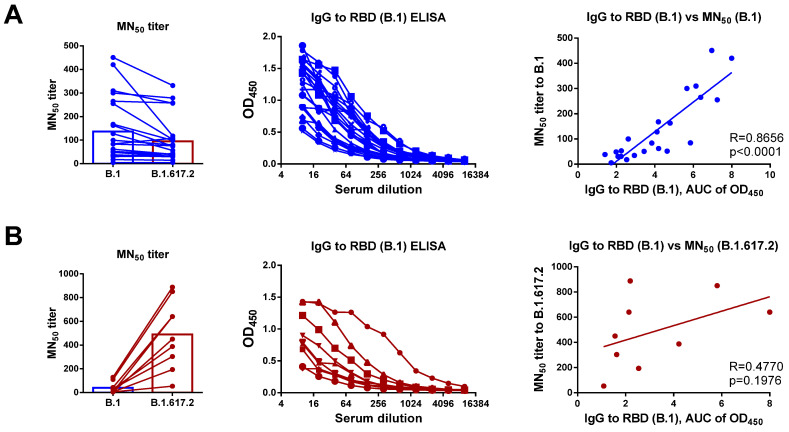
Characterization of virus-neutralizing and RBD-binding antibody levels in COVID-19 convalescents. Serum samples were collected from subjects who recovered from B.1 (Wuhan) SARS-CoV-2 (**A**) and B.1.617.2 (Delta) variant (**B**). Left panel shows MN_50_ titers for B.1 and B.1.617.2 variants of each sample. Middle panel shows ELISA data for RBD (B.1) antigen for each participant. Right panel shows the correlation between MN_50_ titers and RBD-specific IgG levels. The dataset included sera from patients who recovered from B.1 (*n* = 23, blue symbols) and B.1.617.2 (*n* = 9, dark red symbols) variants.

This study was designed to assess the feasibility of using SARS-CoV-2 proteins as antigens in standard antibody tests in the context of constant viral evolution. Therefore, our participants were divided into two cohorts: The first group was infected with the ancestral B.1 (Wuhan D614G) lineage virus (n = 13, with 23 samples collected at different time PSOs), based on the surveillance data [20], while the second group encountered B.1.617.2 (Delta) variant (n = 9, one sample from each subject), as confirmed by Sanger sequencing of the PCR-positive nasal swab specimens or cultured virus (Table S1). Interestingly, sera collected from patients who recovered from the original B.1 SARS-CoV-2 strain had a substantial cross-neutralizing activity against the B.1.617.2 variant (Figure 1A). In contrast, Delta infection raised the antibody levels with a narrow neutralizing activity, as almost no inhibition of Wuhan virus was noted in all studied samples, whereas high homologous neutralizing titers were detected (Figure 1B). Since these two viruses differ significantly by their RBD structures, we compared the RBD-binding activity of serum IgG antibodies in the two cohorts using the available recombinant RBD protein, which corresponds to the sequence of ancestral SARS-CoV-2 virus. As expected, the RBD-binding antibody levels in subjects who recovered from the Wuhan virus correlated well with the B.1 neutralizing capacity of the sera (Figure 1A), whereas no correlation was observed in samples of Delta-recovered patients (Figure 1B). Although the RBD protein corresponding to the B.1.617.2 SARS-CoV-2 strain was not available in our study to prove the correlation of RBD-binding IgG levels with the anti-B.1.617.2 MN_50_ levels in Delta-recovered patients, the low reactivity of their serum with the RBD protein of the B.1 strain suggest that RBD-targeting serology assays developed for the ancestral SARS-CoV-2 virus might not be suitable tests for the detection of a recent infection with antigenically evolved SARS-CoV-2 variants.

To understand whether the N-based antibody tests which were developed for the original SARS-CoV-2 virus are still suitable for the detection of virus-specific antibodies raised to other SARS-CoV-2 variants, we expressed recombinant N proteins from five SARS-CoV-2 lineages and established an in-house ELISA protocol to measure the magnitude of N-specific IgG responses. To assess the specificity of the N protein-based ELISAs, we also expressed the N protein of a seasonal human betacoronavirus strain HCoV-OC43. The protocol for N protein expression was optimized by adding different protein synthesis inducer concentrations (IPTG) and varying the induction time, and the best yield was reached at 0.1 mM IPTG concentration with effective protein production already after 1 h of incubation (Figure S1a). The average yield of recombinant proteins was about 1.2 mg per 100 mL of culture volume. The electrophoretic mobility of proteins corresponded to the predicted molecular weights (~48 kDa). SDS-PAGE of the target fraction demonstrated the absence of significant amounts of contaminating components (Figure S1b). All recombinant proteins were detected by immunoblotting using an anti-Histag probe (Figure S1c). The recombinant N proteins were reliably identified by mass spectrometry based on the obtained spectra, and peptides that unambiguously distinguish the strains from each other were found (Figures S2–S8). In all cases, the sequence coverage was uniform and almost complete, indicating that the analyzed proteins were presented in the samples in the full-length forms (Tables S2–S7).

**Figure 2:**
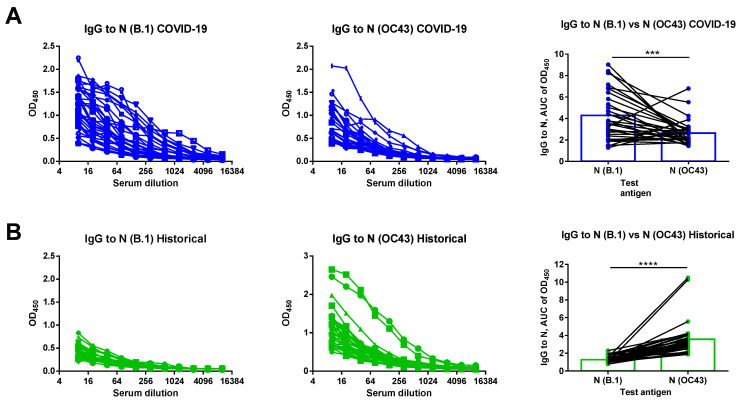
N-based ELISA of serum samples of COVID-19 convalescents (**A**) or historical specimens (**B**). Left panel shows OD_50_ curves for N (B.1) antigen for each participant. Middle panel shows OD_50_ curves for N (OC43) antigen for each participant. Right panel shows comparison of the area under the OD_450_ curve (AUC) values for the two N antigens. The dataset included sera from patients who recovered from COVID-19 (*n* = 32, blue symbols) and historical serum samples (*n* = 36, green symbols). The data were compared by the Wilcoxon matched pairs test. *** *p* < 0.001; **** *p* < 0.0001.

We used the recombinant N (B.1) and N (OC43) proteins as antigens in ELISA and titrated serum samples of COVID-19 convalescents and the historical samples with subsequent calculation of the AUC of OD_450_ values. Interestingly, COVID-19-recovered subjects had high levels of N (B.1)-specific IgG antibodies, as well as significant levels of antibodies binding to the seasonal human coronavirus OC43 antigen (Figure 2A). Given that human coronaviruses widely circulated in Saint Petersburg in the 2020/2021 season, most probably some of our patients have contracted seasonal HCoV infection after recovery from SARS-CoV-2 [21]. In contrast, historical samples were characterized by strong reactivity with the seasonal coronavirus N antigen, but not with the N (B.1) protein (Figure 2B). Some level of B.1-specific IgG antibodies in this study group can be explained by the cross-reactivity of N-specific antibody between different coronaviruses or by non-specific binding of the some other human antibodies to N protein.

**Figure 3:**
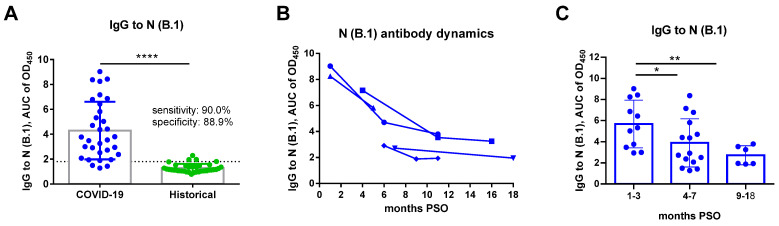
N-based ELISA of serum samples of COVID-19 convalescents and historical samples. (**A**) The area under the OD_450_ curve (AUC) values were compared between COVID-19 patients and historical samples to assess the specificity and sensitivity of the developed in-house N-based ELISA. Data were compared by the two-sided Mann-Whitney U test. (**B**) The dynamics of N-specific antibody were analyzed for the selected patients. (**C**) The AUC values of COVID-19 patients grouped by the time post symptoms onset (PSO). Data were compared by one-way ANOVA with Tukey’s multiple comparisons test. The dataset included sera from patients who recovered from COVID-19 (*n* = 32, blue symbols) and historical serum samples (*n* = 36, green symbols). * *p* < 0.05; ** *p* < 0.01; **** *p* < 0.0001.

Nonetheless, when the N (B.1) protein was used in ELISA, titration of serum samples and calculating the AUC of OD_450_ values could reliably identify the COVID-19 cases with sensitivity of 90.0% and specificity of 88.9%, when the cut-off AUC value was set as 1.8 (Figure 3A). The N-specific antibody waned over time, which was in line with other studies [22,23], suggesting that the sensitivity of our in-house ELISA may decrease with the increasing time of PSO (Figure 3B,C). Nevertheless, the established specificity of the ELISA in detecting SARS-CoV-2 N (B.1)-targeted serum IgG antibodies in COVID-19 patient sera allows the testing of the cross-reactivity of these antibodies against other SARS-CoV-2 lineages.

**Figure 4:**
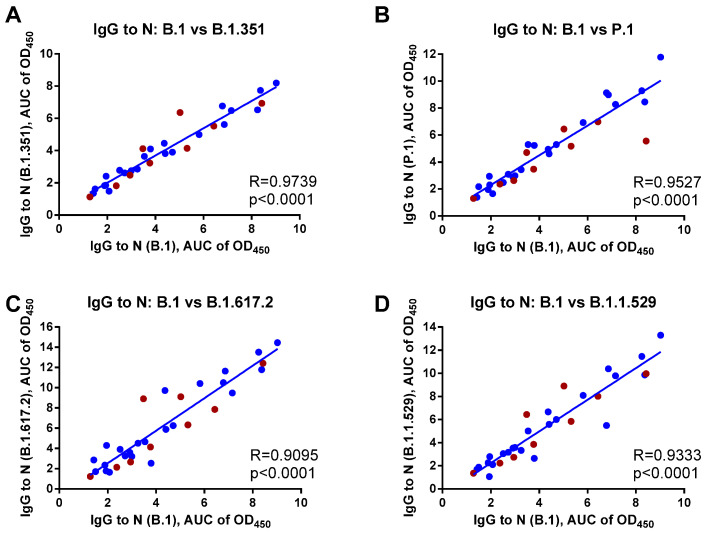
Correlation analyses of N-specific IgG antibody levels in COVID-19 convalescents between the N protein of B.1 (Wuhan) strain and (**A**) B.1.351 (Beta); (**B**) P.1 (Gamma); (**C**) B.1.617.2 (Delta); and (**D**) B.1.1.529 (Omicron) variants. Sera from patients who recovered from B.1 (blue dots) and B.1.617.2 (dark red dots) variants were analyzed (*n* = 32).

Next, we assessed the cross-reactivity of the N-targeted antibodies in sera of COVID-19 convalescents against N proteins of four SARS-CoV-2 variants: B.1.351 (Beta), P. (Gamma), B.1.617.2 (Delta), and B.1.1.529 (Omicron) and found a strong positive correlation with the antibody against the N protein of ancestral B.1 (Wuhan) virus, with R values ranging from 0.9095 to 0.9739, all *p* < 0.0001 (Figure 4). This analysis included both patient subsets, with no significant differences found between the cross-reactive potential of the anti-N IgG antibody raised to Wuhan or Delta SARS-CoV-2 infection. Overall, these data justify the suitability of the approved N-based serology assays developed on the basis of the ancestral SARS-CoV-2 virus for monitoring coronavirus infections caused by antigenically distant SARS-CoV-2 variants.

## 4. Discussion

SARS-CoV-2 infection triggers various pathological changes in the host immune system [24], and depending on the severity of the disease and the individual immune status of the infected individual, a humoral immune response can develop predominantly to the Spike protein or to the nucleocapsid [9,25,26]. Measuring virus-specific antibody levels is a relatively quick and inexpensive procedure to assess the status of the disease, the probability of reinfection, and the immunogenicity and the longevity of COVID-19 vaccine-induced immune responses [27,28,29,30]. Due to the functional activity of the receptor-binding domain of the Spike protein, the level of anti-RBD antibody is generally considered as a surrogate marker of the viral neutralizing potential of a patient’s serum. Therefore, RBD-based serology tests are widely used in clinical practice [31,32,33,34]. However, the RBD is one of the most mutation-prone viral antigens, since the epitopes in RBD undergo rapid evolution to escape binding with polyclonal antibodies, which is raised to prior infection or vaccination with Spike-based vaccines [35,36,37]. As a result, the antibody levels to the RBD of the ancestral SARS-CoV-2 strain may no longer predict the neutralizing capacity against other variants of concern. Indeed, we herein confirmed that the RBD-binding IgG antibody strongly correlates with MN_50_ titers against the homologous B.1 lineage virus, but not against the B.1.617.2 variant, suggesting that the RBD-based serology tests should be timely updated based on the antigenic properties of the newly emerging SARS-CoV-2 variants.

SARS-CoV-2 N protein is another frequently used antigen in serology testing of COVID-19 in the community. In addition, it is important to monitor N-specific IgG antibody levels in studies of the longevity of vaccine-induced immune responses, since some subjects with re-infection during the monitoring period can be detected by the increase in N-specific antibodies and should be excluded from the analyses [38]. For example, in a study by Gonzalez Lopez Ledesma et al., an increased cross-neutralization activity over time for variants of concern (VOCs) was observed in Sputnik V recipients that were naïve at baseline [39]. However, the naïve status was monitored only by the presence of anti-Spike antibodies, without further testing of serum specimens for anti-N antibodies to identify a possible natural infection within the study cohort during the trial. Since the study took place at the time of active virus circulation, there was a possibility to overlook the COVID-19 cases that could have influenced the results of longitudinal studies. This assumption is supported by the findings that significantly lower neutralizing titers to some VOCs compared to the ancestral SARS-CoV-2 strain were observed in vaccinated seronegative individuals. However, this difference was not significant in the vaccines that have been previously diagnosed with COVID-19 [40,41].

Although the N protein is more conserved compared to Spike [42], the impact of the N mutations on the performance of the approved antibody tests has not yet been thoroughly studied [43]. Here, we assessed the cross-reactivity of N-specific antibodies in COVID-19 patients by establishing an in-house ELISA protocol based on the recombinant N proteins of different SARS-CoV-2 VOCs. It is known that expression in *E. coli* and purification of His-tagged proteins by IMAC is a cost-effective and versatile method. Indeed, we were able to obtain about 1 mg of the N protein per 100 mL of bacterial culture. Importantly, the COVID-19 convalescent sera could specifically recognize the SARS-CoV-2 N protein, while the reactivity of the same samples with the N protein of seasonal coronavirus can be explained by previous infections, rather than by the cross-reactivity of N-specific antibodies between SARS-CoV-2 and HCoVs. Moreover, we noted a significant decrease in N-specific antibody 1 year after the infection, which is in agreement with other studies [44], and these findings indicate that the N-based test systems may have reduced sensitivity with the increasing time of PSO. The limitation of our assay is that N-specific IgG standards for each SARS-CoV-2 strain were not available to estimate the precise concentrations of N-binding antibodies in convalescent sera, and further studies using a larger set of samples are needed to clarify these values. Nevertheless, we found a strong positive correlation in the magnitude of anti-N (B.1) antibodies and antibodies specific to four other VOCs in COVID-19-recovered patients, suggesting that the N-binding antibodies are highly cross-reactive, and the most immunogenic epitopes within this protein are not under selective pressure.

Overall, our study suggests that the RBD-based serology tests should be timely updated to reflect the constantly evolving nature of the SARS-CoV-2 Spike protein, whereas the N-based test systems developed using the N protein of the ancestral (Wuhan) virus are suitable for the analysis of sera from COVID-19 patients regardless of the strain that caused the infection. Further studies with validated serological tests are needed to confirm our findings.

## Data Availability

The data presented in this study are available on request from the corresponding author.

## Supplementary Materials

The following supporting information can be downloaded at: https://www.mdpi.com/article/10.3390/v14092041/s1. Figure S1: Induction and purification of recombinant N proteins. Figure S2: Comparison of amino acid sequences of recombinant N proteins based on the results of mass spectrometric study. Figure S3: Mass spectrometry analysis of N protein (B.1). Figure S4: Mass spectrometry analysis of N protein (B.1.351). Figure S5: Mass spectrometry analysis of N protein (P.1). Figure S6: Mass spectrometry analysis of N protein (B.1.617.2). Figure S7: Mass spectrometry analysis of N protein (B.1.1.529). Figure S8: Mass spectrometry analysis of N protein (HCoV OC43). Table S1: Demographic characteristics of the study subjects. Table S2: Detected ions (MH+) and matched peptides from the recombinant N protein (B.1) according to peptide mapping. Table S3: Detected ions (MH+) and matched peptides from the recombinant N protein (B.1.351) according to peptide mapping. Table S4: Detected ions (MH+) and matched peptides from the recombinant N protein (P.1) according to peptide mapping. Table S5: Detected ions (MH+) and matched peptides from the recombinant N protein (B.1.617.2) according to peptide mapping. Table S6: Detected ions (MH+) and matched peptides from the recombinant N protein (B.1.1.529) according to peptide mapping. Table S7: Detected ions (MH+) and matched peptides from the recombinant N protein (HCoV OC43) according to peptide mapping. Reference [20] are cited in the supplementary materials.
