# Supplementary material for: Cross-Reactivity of SARS-CoV-2 Nucleocapsid-Binding Antibodies and Its Implication for COVID-19 Serology Tests
Source: Viruses. 2022 Sep 14;14(9):2041. doi: 10.3390/v14092041 (PMC9502088; doi:10.3390/v14092041)
Supplement: Supplementary file 1 [file viruses-14-02041-s001.zip › viruses-1856674-supplementary.pdf]

viruses

MDPI

Supplementary Materials

Table S1. Demographic characteristics of the study subjects

№	Sex	Age	Date of disease onset	SARS-CoV-2 lineage†	Severity	Month PSO
1	F	31	May 2020	B.1	severe	7
2	F	31	May 2020	B.1	severe	18
3	M	39	May 2020	B.1	severe	4
4	M	39	May 2020	B.1	severe	11
5	M	39	May 2020	B.1	severe	16
6	F	34	May 2020	B.1	mild	7
7	F	40	June 2020	B.1	mild	6
8	M	38	Sep 2020	B.1	moderate	2
9	F	33	Oct 2020	B.1	moderate	6
10	F	33	Oct 2020	B.1	moderate	9
11	F	33	Oct 2020	B.1	moderate	11
12	F	77	Oct 2020	B.1	moderate	1
13	F	77	Oct 2020	B.1	moderate	6
14	F	77	Oct 2020	B.1	moderate	11
15	F	39	Nov 2020	B.1	mild	6
16	M	39	Dec 2020	B.1	moderate	1
17	M	39	Dec 2020	B.1	moderate	5
18	M	39	Dec 2020	B.1	moderate	7
19	F	39	Dec 2020	B.1	mild	5
20	F	39	Feb 2021	B.1	mild	1
21	F	39	Feb 2021	B.1	mild	6
22	F	80	Feb 2021	B.1	moderate	7
23	M	84	Feb 2021	B.1	severe	7
24	F	64	May 2021	B.1.617.2	mild	1
25	F	37	May 2021	B.1.617.2	mild	4
26	M	37	May 2021	B.1.617.2	moderate	4
27	F	45	May 2021	B.1.617.2	mild	1
28	M	26	Aug 2021	B.1.617.2	mild	1
29	M	31	Aug 2021	B.1.617.2	mild	1
30	F	38	Sep 2021	B.1.617.2	mild	1
31	M	31	Oct 2021	B.1.617.2	mild	1
32	F	35	Oct 2021	B.1.617.2	mild	1

† the B.1 (Wuhan D614G) lineage was assigned based on the surveillance data [1], while B.1.617.2 (Delta) lineage assignment was based on the sequencing of Spike protein gene of the RT-PCR positive specimens or recovered live viruses.
PSO – post symptoms onset.

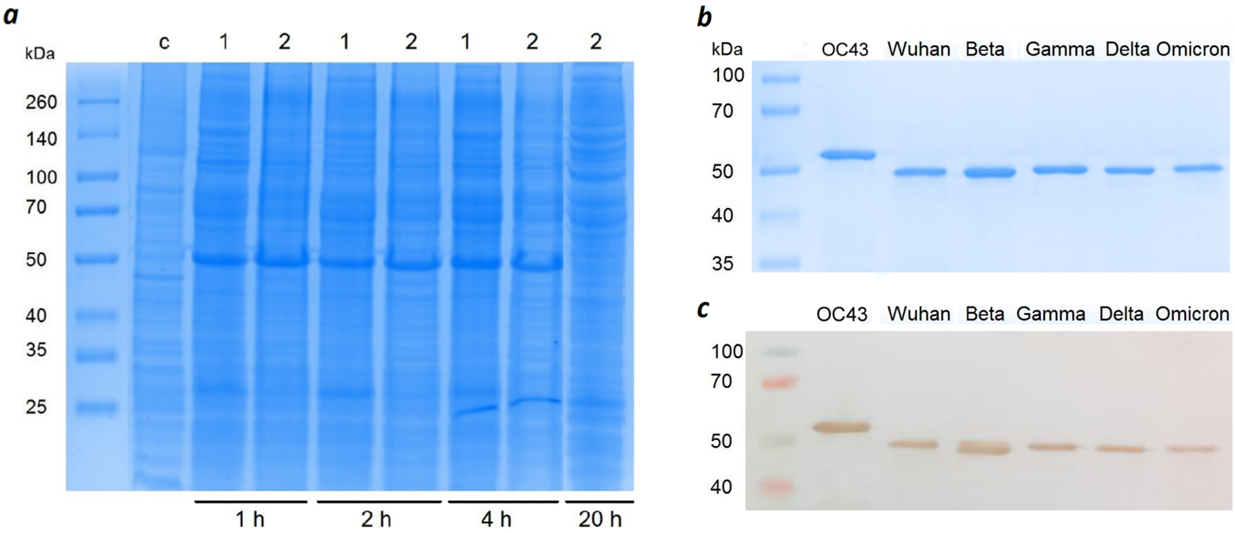

Figure S1. Induction and purification of recombinant N proteins. (**a**) SDS-PAGE analysis of different IPTG concentrations and induction time on the expression intensity of N protein (B.1) at 37 °C (1, 0.1 mM IPTG; 2, 0.5 mM IPTG); (**b**) SDS-PAGE analysis of N proteins purified by IMAC under non-reducing conditions; (**c**) Western blot analysis of obtained proteins with HisProbe-HRP. M - molecular weight marker.

A

```
                10         20         30         40         50         60         70         80         90        100
1 :  MGSSHHHHHH SQDQMSDNGP QNQRNAPRIT FGGPSDSTGS NQNGERSGAR SKQRRPQGLP NNTASWFTAL TQHGKEDLKF PRGQGVPINT NSSPDDQIGY
2 :  MGSSHHHHHH SQDQMSDNGP QNQRNASRIT FGGPSDSTGS NQNGERSGAR SKQRRPQGLP NNTASWFTAL TQHGKEDLKF PRGQGVPINT NSSPDDQIGY
3 :  MGSSHHHHHH SQDQMSDNGP QNQRNAPRIT FGGPSDSTGS NQNGERSGAR SKQRRPQGLP NNTASWFTAL TQHGKEDLKF PRGQGVPINT NSSRDDQIGY
4 :  MGSSHHHHHH SQDQMSDNGP QNQRNAPRIT FGGPSDSTGS NQNGERSGAR SKQRRPQGLP NNTASWFTAL TQHGKEGLKF PRGQGVPINT NSSPDDQIGY
5 :  MGSSHHHHHH SQDQMSDNGP QNQRNALRIT FGGPSDSTGS NQNG---GAR SKQRRPQGLP NNTASWFTAL TQHGKEDLKF PRGQGVPINT NSSPDDQIGY

               110        120        130        140        150        160        170        180        190        200
     YRRATRRIRG GDGKMKDLSP RWYFYYLGTG PEAGLPYGAN KDGIIWVATE GALNTPKDHI GTRNPANNAA IVLQLPQGTT LPKGFYAEGS RGGSQASSRS
     YRRATRRIRG GDGKMKDLSP RWYFYYLGTG PEAGLPYGAN KDGIIWVATE GALNTPKDHI GTRNPANNAA IVLQLPQGTT LPKGFYAEGS RGGSQASSRS
     YRRATRRIRG GDGKMKDLSP RWYFYYLGTG PEAGLPYGAN KDGIIWVATE GALNTPKDHI GTRNPANNAA IVLQLPQGTT LPKGFYAEGS RGGSQASSRS
     YRRATRRIRG GDGKMKDLSP RWYFYYLGTG PEAGLPYGAN KDGIIWVATE GALNTPKDHI GTRNPANNAA IVLQLPQGTT LPKGFYAEGS RGGSQASSRS
     YRRATRRIRG GDGKMKDLSP RWYFYYLGTG PEAGLPYGAN KDGIIWVATE GALNTPKDHI GTRNPANNAA IVLQLPQGTT LPKGFYAEGS RGGSQASSRS

               210        220        230        240        250        260        270        280        290        300
     SSRSRNSSRN STPGSSRGTS PARMAGNGGD AALALLLLDR LNQLESKMSG KGQQQQGQTV TKKSAAEASK KPRQKRTATK AYNVTQAFGR RGPEQTQGNF
     SSRSRNSSRN STPGSSRGIS PARMAGNGGD AALALLLLDR LNQLESKMSG KGQQQQGQTV TKKSAAEASK KPRQKRTATK AYNVTQAFGR RGPEQTQGNF
     SSRSRNSSRN STPGSSKRTS PARMAGNGGD AALALLLLDR LNQLESKMSG KGQQQQGQTV TKKSAAEASK KPRQKRTATK AYNVTQAFGR RGPEQTQGNF
     SSRSRNSSRN STPGSSMGTS PARMAGNGGD AALALLLLDR LNQLESKMSG KGQQQQGQTV TKKSAAEASK KPRQKRTATK AYNVTQAFGR RGPEQTQGNF
     SSRSRNSSRN STPGSSKRTS PARMAGNGGD AALALLLLDR LNQLESKMSG KGQQQQGQTV TKKSAAEASK KPRQKRTATK AYNVTQAFGR RGPEQTQGNF

               310        320        330        340        350        360        370        380        390        400
     GDQELIRQGT DYKHWPQIAQ FAPSASAFFG MSRIGMEVTP SGTWLTYTGA IKLDDKDPNF KDQVILLNKH IDAYKTFPPT EPKKDKKKKA DETQALPQRQ
     GDQELIRQGT DYKHWPQIAQ FAPSASAFFG MSRIGMEVTP SGTWLTYTGA IKLDDKDPNF KDQVILLNKH IDAYKTFPPT EPKKDKKKKA DETQALPQRQ
     GDQELIRQGT DYKHWPQIAQ FAPSASAFFG MSRIGMEVTP SGTWLTYTGA IKLDDKDPNF KDQVILLNKH IDAYKTFPPT EPKKDKKKKA DETQALPQRQ
     GDQELIRQGT DYKHWPQIAQ FAPSASAFFG MSRIGMEVTP SGTWLTYTGA IKLDDKDPNF KDQVILLNKH IDAYKTFPPT EPKKDKKKKA YETQALPQKQ
     GDQELIRQGT DYKHWPQIAQ FAPSASAFFG MSRIGMEVTP SGTWLTYTGA IKLDDKDPNF KDQVILLNKH IDAYKTFPPT EPKKDKKKKA DETQALPQRQ

               410        420        430              Cov    Score
     KKQQTVTLLP AADLDDFSKQ LQQSMSSADS TQAHHHHHH    87%   330 / 70
     KKQQTVTLLP AADLDDFSKQ LQQSMSSADS TQAHHHHHH    85%   243 / 70
     KKQQTVTLLP AADLDDFSKQ LQQSMSSADS TQAHHHHHH    72%   339 / 70
     KKQQTVTLLP AADLDDFSKQ LQQSMSSADS TQAHHHHHH    71%   238 / 70
     KKQQTVTLLP AADLDDFSKQ LQQSMSSADS TQAHHHHHH    86%   267 / 70
```

1 – hCoV-19/Russia/StPetersburg-3524/2020 (B.1 Lineage, Wuhan)
2 – hCoV-19/Russia/SPE-RII-27029S/2021 (B.1.351 Lineage, Beta)
3 – hCoV-19/Japan/TY7-503/2021 (P.1 Lineage, Gamma)
4 – hCoV-19/Russia/SPE-RII-32759S/2021 (B.1.617.2 Lineage, Delta)
5 – hCoV-19/Russia/SPE-RII-6243V1/2021 (B.1.1.529 Lineage, Omicron)
S – hCoV_OC43

B

```
                10         20         30         40         50         60         70         80         90        100
1 :  MGSSHHHHHH SQDQMS---- ---------D NGPQNQRNAP RITFGGPSDS TGSNQNGERS GARSKQRRPQ GLPNNTASWF TALTQHGK-E DLKFPRGQGV
S :  MGSSHHHHHH SQDQMSFTPG KQSSSRASSG NRSGNGILKW ADQSDQVRNV QTRGRRAQPK QTATSQQPSG GNVVPYYSWF SGITQFQKGK EFEFVEGQGV

               110        120        130        140        150        160        170        180        190        200
     PINTNSSPDD QIGYYRRATR R-IRGGDGKM KDLSPRWYFY YLGTGPEAGL PYGANKDGII WVATEGA-LN TPKDHIGTRN PANNAAIVLQ LPQGTTLPKG
     PIAPGVPATE AKGYWYRHNR RSFKTADGNQ RQLLPRWYFY YLGTGPHAKD QYGTDIDGVY WVASNQADVN TPAD-IVDRD PSSDEAIPTR FPPGTVLPQG
               210        220        230        240        250        260        270        280        290        300
     FYAEGS---- ------RGGS QASSRSSSRS RNSSRNSTPG SSRGTSPARM AGNGGDAALA LLLLDRLNQL ESKMSGKGQQ QQGQTVTKKS AAEASK----
     YYIEGSGRSA PNSRSTSRTS SRASSAGSRS RANSGNRTPT S--GVTPD-M ADQ-----IA SLVLAKLGKD ATK------- --PQQVTKHT AKEVRQKILN

               310        320        330        340        350        360        370        380        390        400
     KPRQKRTATK AYNVTQAFGR RGPEQTQGNF GDQELIRQGT DYKHWPQIAQ FAPSASAFFG MSRIGM---- -------EVT PSGTWLTYTG AIKLDDKDPN
     KPRQKRSPNK QCTVQQCFGK RGPNQ---NF GGGEMLKLGT SDPQFPILAE LAPTAGAFFF GSRLELAKVQ NLSGNPDEPQ KDVYELRYNG AIRFDSTLSG

               410        420        430        440        450        460        470        480              Cov    Score
     FKDQVILLNK HIDAYKTFPP TEPKKDKKKK ADETQALPQR QKKQQTVTLL PAADLDDFSK QLQQSMSSAD STQA------ ---HHHHHH    87%   330 / 70
     FETIMKVLNE NLNAYQQQDG MMNMSPKPQR QRGHKNGQGE NDNISVAVPK SRVQQNKSRE LTAEDISLLK KMDEPYTEDT SEIHHHHHH    79%   316 / 70
```

Figure S2. Comparison of amino acid sequences of recombinant N proteins based on the results of mass spectrometric study. **A.** Alignment of five sequences of SARS-CoV-2 strains used in the study. **B.** Comparison of the N proteins sequences of B.1 SARS-CoV-2 (1) and a seasonal OC43 (*S*) coronavirus. Identified amino acid residues are marked in red. Yellow and blue colors indicate residues that differ in the analyzed proteins. Areas that distinguish at least one of the proteins are marked in bold red, and unique regions allowing to unambiguously identify the proteins are underlined. Unique regions that distinguish the N proteins of SARS-CoV-2 and OC43 viruses are shown in frames. Coverage and identification score (value/threshold) for each protein are indicated; the identification is considered reliable (p<0.05) if the score exceeds the threshold.

A

B

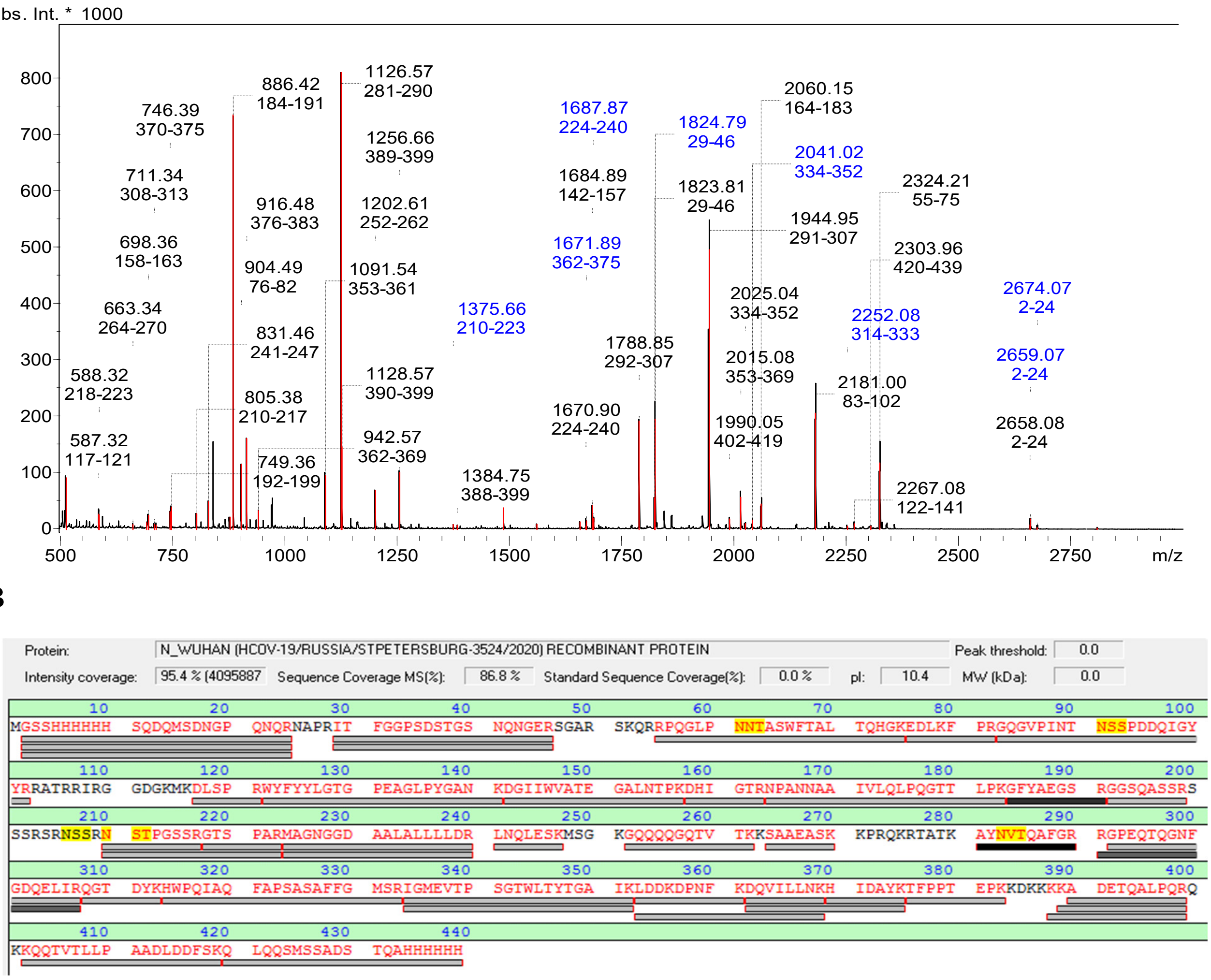

Figure S3. Mass spectrometry analysis of N protein (B.1). **A.** Fragment of MALDI mass spectrum of hydrolyzed protein. Ions associated with fragments of the amino acid sequence (hCoV-19/Russia/StPetersburg-3524/2020) and the corresponding ranges are indicated; **B.** The amino acid sequence of protein: the fragments found in the spectrum are marked in red, gray underlined regions are matched with registered ions.

A

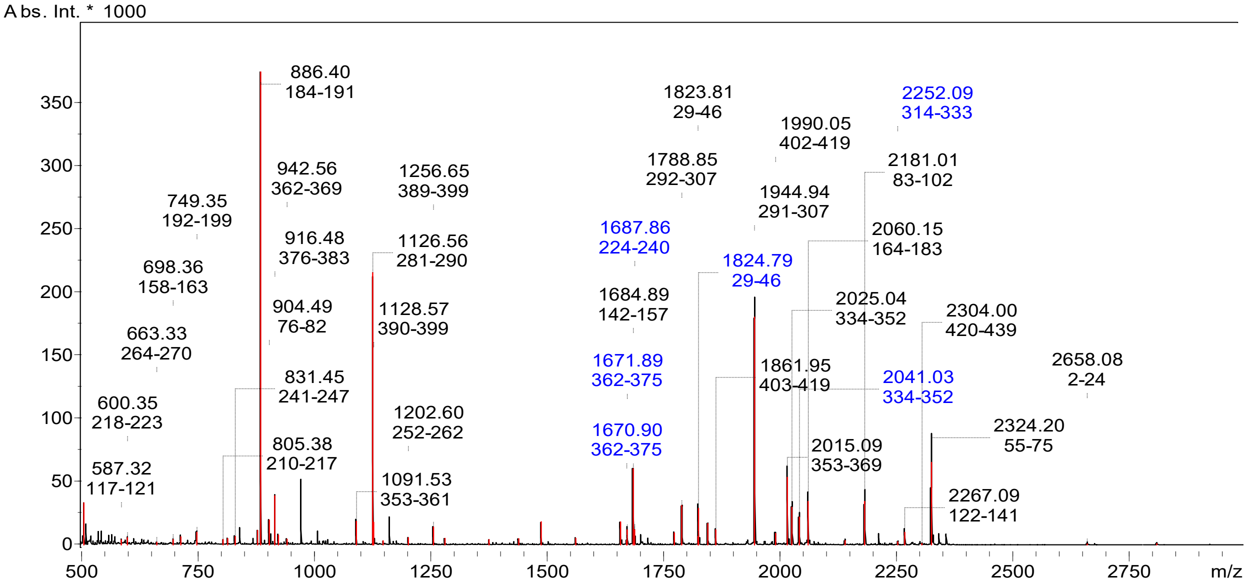

B

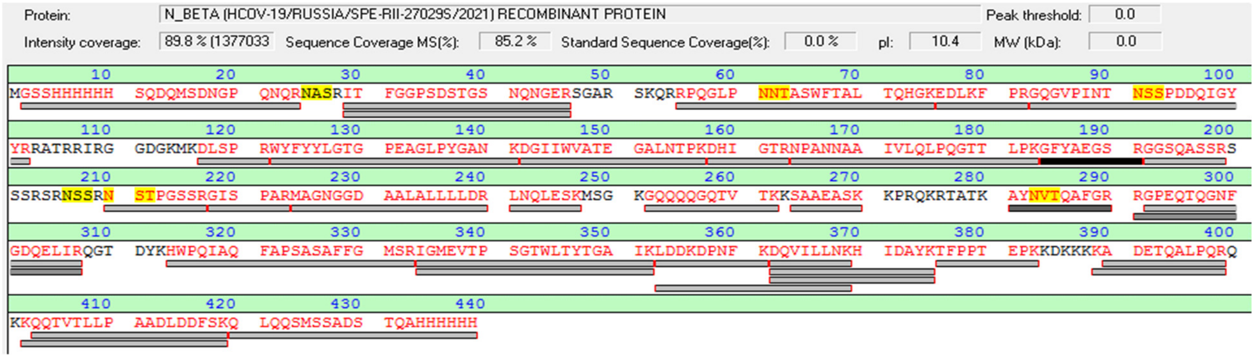

Figure S4. Mass spectrometry analysis of N protein (B.1.351). **A.** Fragment of MALDI mass spectrum of hydrolyzed protein. Ions associated with fragments of the amino acid sequence (hCoV-19/Russia/SPE-RII-27029S/2021) and the corresponding ranges are indicated; **B.** The amino acid sequence of protein: the fragments found in the spectrum are marked in red, gray underlined regions are matched with registered ions.

A

B

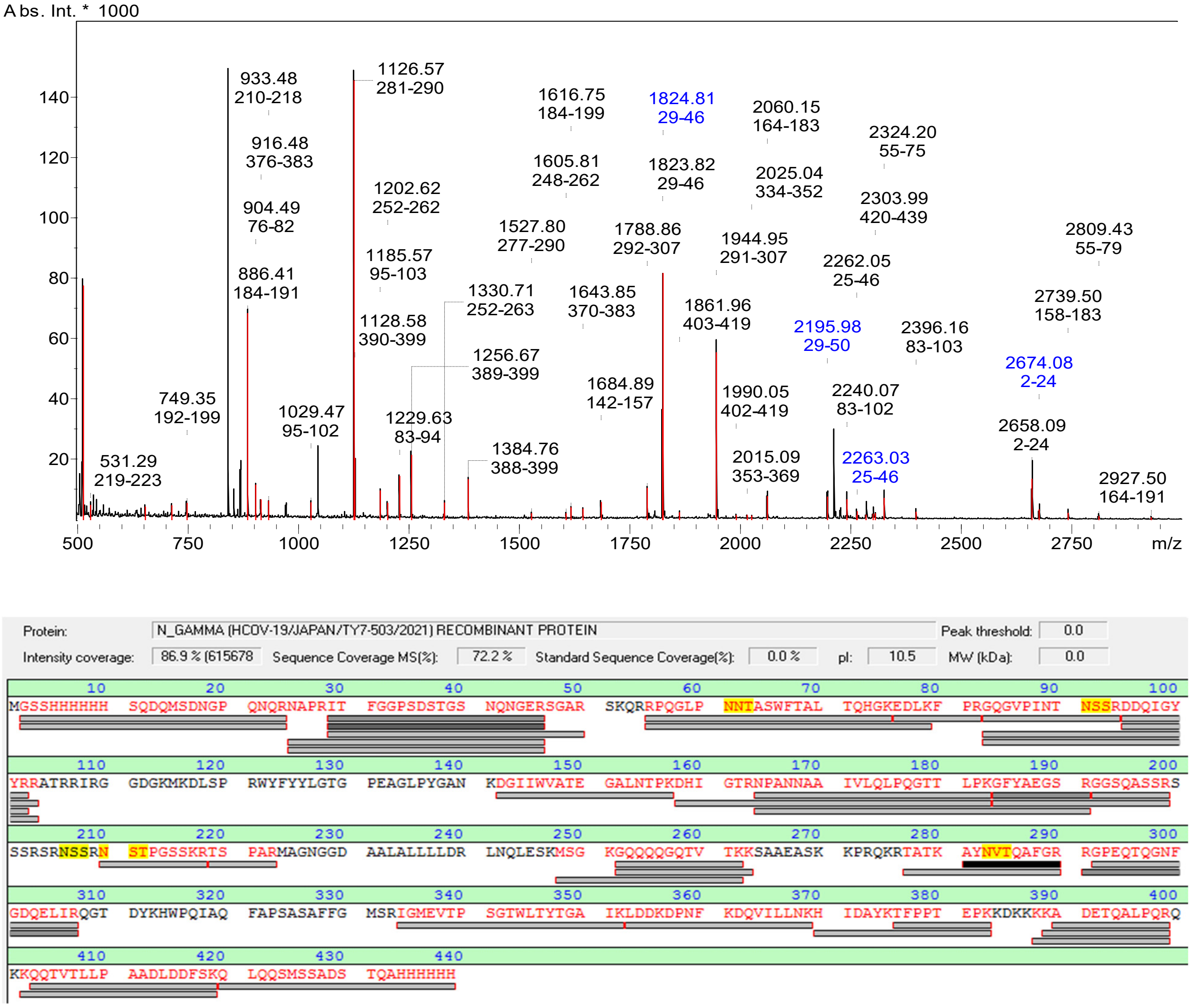

Figure S5. Mass spectrometry analysis of N protein (P.1). **A.** Fragment of MALDI mass spectrum of hydrolyzed protein. Ions associated with fragments of the amino acid sequence (hCoV-19/Japan/TY7-503/2021) and the corresponding ranges are indicated; **B.** The amino acid sequence of protein: the fragments found in the spectrum are marked in red, gray underlined regions are matched with registered ions.

A

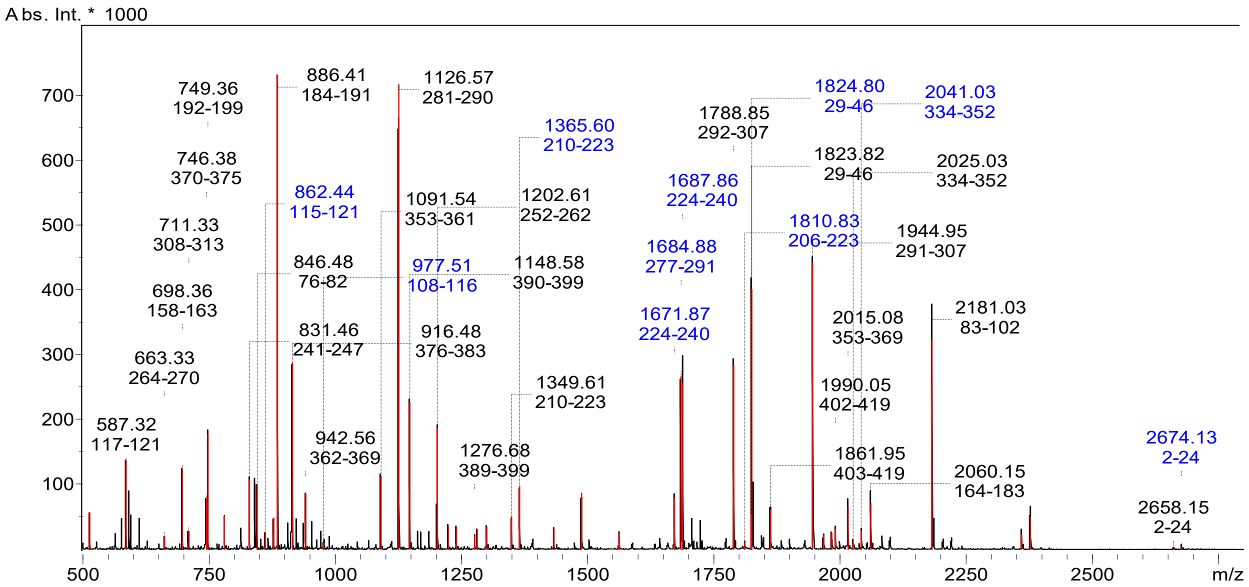

B

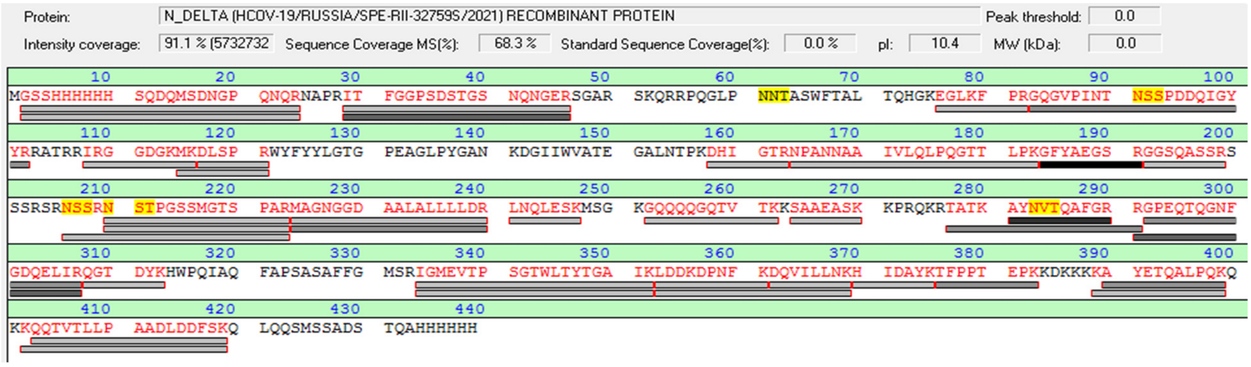

Figure S6. Mass spectrometry analysis of N protein (B.1.617.2). **A.** Fragment of MALDI mass spectrum of hydrolyzed protein. Ions associated with fragments of the amino acid sequence (hCoV-19/Russia/SPE-RII-32759S/2021) and the corresponding ranges are indicated; **B.** The amino acid sequence of protein: the fragments found in the spectrum are marked in red, gray underlined regions are matched with registered ions.

A

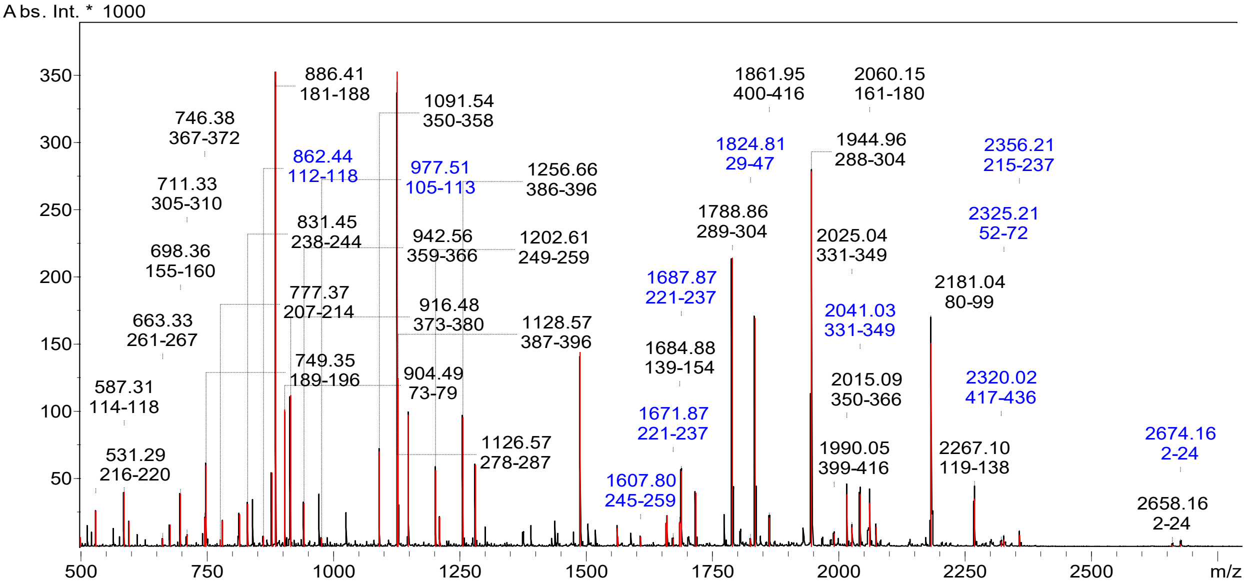

B

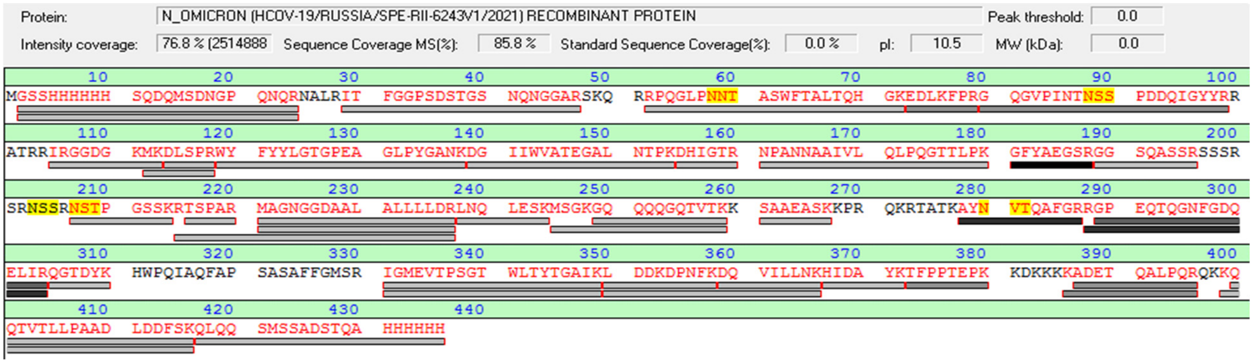

Figure S7. Mass spectrometry analysis of N protein (B.1.1.529). **A.** Fragment of MALDI mass spectrum of hydrolyzed protein. Ions associated with fragments of the amino acid sequence (hCoV-19/Russia/SPE-RII-6243V1/2021) and the corresponding ranges are indicated; **B.** The amino acid sequence of protein: the fragments found in the spectrum are marked in red, gray underlined regions are matched with registered ions.

A

B

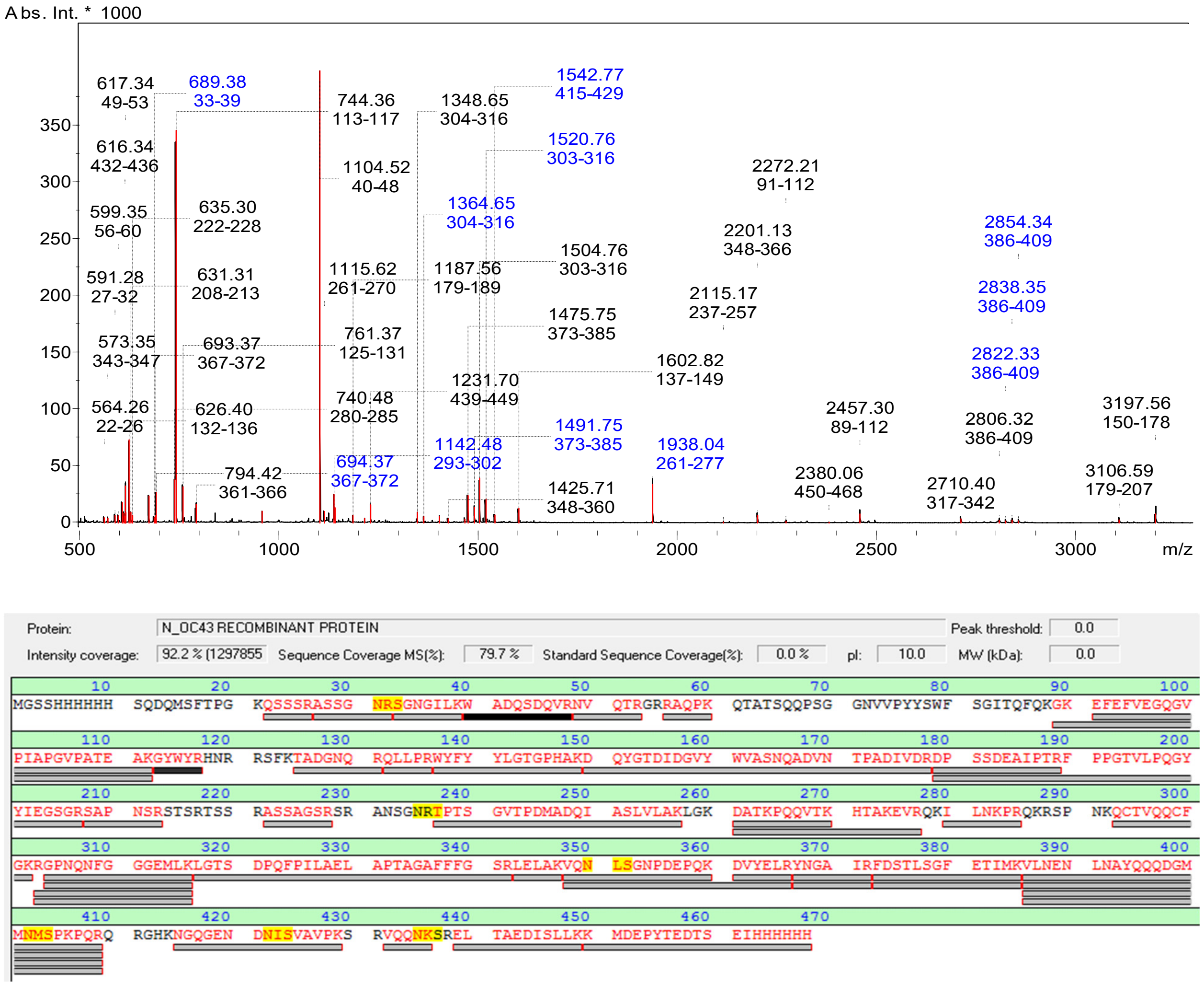

Figure S8. Mass spectrometry analysis of N protein (HCoV OC43). **A.** Fragment of MALDI mass spectrum of hydrolyzed protein. Ions associated with fragments of the amino acid sequence (OC43) and the corresponding ranges are indicated; **B.** The amino acid sequence of protein: the fragments found in the spectrum are marked in red, gray underlined regions are matched with registered ions.

Table S2. Detected ions (MH+) and matched peptides from the recombinant N protein (B.1) according to peptide mapping. The range of residues corresponding to the peptides, the relative intensity of the ion, the mass deviation (in parts per million, ppm), as well as possible peptide modifications (oxidation and deamidation) are indicated. Peptides that differ in at least one of the studied N proteins are marked in red, and unique peptides for this recombinant proteins are marked in bold red.

MH^+	Intensity (RU)	Mass deviation (ppm)	Range of residues	Matched peptide
587.31	27512.69	11.37	117 - 121	DLSPR
**588.31**	**25817.61**	**9.47**	**218 - 223**	**GTSPAR**
663.33	8914.68	12.56	264 - 270	SAAEASK
698.36	25481.97	6.72	158 - 163	DHIGTR
711.33	9493.81	10.66	308 - 313	QGTDYK
746.38	31731.21	6.74	370 - 375	HIDAYK
749.35	39593.38	3.67	192 - 199	GGSQASSR
**805.38**	**28000.48**	**1.61**	**210 - 217**	**NSTPGSSR**
831.46	49533.04	6.3	241 - 247	LNQLESK
886.41	733180.94	12.52	184 - 191	GFYAEGSR
904.49	115198.42	4.06	76 - 82	EDLKFPR
916.48	160751.78	7.68	376 - 383	TFPPTEPK
942.56	33435.58	3.63	362 - 369	DQVILLNK
1091.54	95118.6	4.06	353 - 361	LDDKDPNFK
1126.56	830947.64	5.7	281 - 290	AYNVTQAFGR
1128.56	145732.46	5.78	390 - 399	ADETQALPQR
1202.61	69095.96	-4.59	252 - 262	GQQQQGQTVTK
1256.66	101407.45	-0.38	389 - 399	KADETQALPQR
**1375.66**	**8647.57**	**-0.21**	**210 - 223**	**NSTPGSSRGTSPAR 1: Deamidated (NQ)**
1384.75	7247.41	-1.36	388 - 399	KKADETQALPQR
1670.89	6429	6.47	224 - 240	MAGNGGDAALALLLLDR
1671.9	15410.38	-5.26	362 - 375	DQVILLNKHIDAYK 2: Deamidated (NQ) 7: Deamidated (NQ)
1684.89	42657.95	1.67	142 - 157	DGIIWVATEGALNTPK
1687.87	19729.67	-1.68	224 - 240	MAGNGGDAALALLLLDR 1: Oxidation (M) 4: Deamidated (NQ)
1788.85	192022.96	1.82	292 - 307	GPEQTQGNFGDQELIR
1823.82	56772	-5.18	29 - 46	ITFGGPSDSTGSNQNGER
1824.8	195305.65	-5.61	29 - 46	ITFGGPSDSTGSNQNGER 13: Deamidated (NQ)
1944.95	495320.52	-1.67	291 - 307	RGPEQTQGNFGDQELIR
1990.05	18602.11	0.26	402 - 419	KQQTVTLLPAADLDDFSK
2015.08	57246.82	-1.74	353 - 369	LDDKDPNFKDQVILLNK
2025.04	10393.14	0.49	334 - 352	IGMEVTPSGTWLTYTGAIK
2041.03	16691.14	-5.29	334 - 352	IGMEVTPSGTWLTYTGAIK 3: Oxidation (M)
2060.15	45208.99	-0.72	164 - 183	NPANNAAIVLQLPQGTTLPK
2181.02	205942.06	-7.23	83 - 102	GQGVPINTNSSPDDQIGYYR

2252.07	5949.45	3.89	314 - 333	HWPQIAQFAPSASAFFGMSR 18: Oxidation (M)
2267.08	10286.59	1.87	122 - 141	WYFYYLGTGPEAGLPYGANK
2304.01	6300	-19.61	420 - 439	QLQQSMSSADSTQAHHHHHH
2324.19	117640.71	8.28	55 - 75	RPQGLPNNTASWFTALTQHGK
2658.11	8524	-11.62	2 - 24	GSSHHHHHHSQDQMSDNGPQNQR
2659.09	18997	-9.85	2 - 24	GSSHHHHHHSQDQMSDNGPQNQR 11: Deamidated (NQ)
2674.11	3614	-14.13	2 - 24	GSSHHHHHHSQDQMSDNGPQNQR 14: Oxidation (M)

Table S3 - Detected ions (MH+) and matched peptides from the recombinant N protein (B.1.351) according to peptide mapping. The range of residues corresponding to the peptides, the relative intensity of the ion, the mass deviation (in parts per million, ppm), as well as possible peptide modifications (oxidation and deamidation) are indicated. Peptides that differ in at least one of the studied N proteins are marked in red, and unique peptides for this recombinant proteins are marked in bold red.

MH^+	Intensity (RU)	Mass deviation (ppm)	Range of residues	Sequence
587.31	4137.63	2.93	117 - 121	DLSPR
**600.35**	**5917.54**	**3.05**	**218 - 223**	**GISPAR**
663.33	2329	3.04	264 - 270	SAAEASK
698.36	4479.49	-0.38	158 - 163	DHIGTR
749.35	10225.64	-1.96	192 - 199	GGSQASSR
**805.38**	**4407.71**	**-1.79**	**210 - 217**	**NSTPGSSR**
831.46	6944.56	-3.12	241 - 247	LNQLESK
886.41	390911.05	-2.2	184 - 191	GFYAEGSR
904.49	19787.71	0.5	76 - 82	EDLKFPR
916.48	38991.55	-2.62	376 - 383	TFPPTEPK
942.56	4561.42	-0.32	362 - 369	DQVILLNK
1091.54	18783.54	-4.54	353 - 361	LDDKDPNFK
1126.56	215077.87	-4.21	281 - 290	AYNVTQAFGR
1128.56	18023.5	6.14	390 - 399	ADETQALPQR
1202.61	5850.88	-8.65	252 - 262	GQQQQGQTVTK
1256.66	14265.01	-5.78	389 - 399	KADETQALPQR
1670.91	3870	-4.73	362 - 375	DQVILLNKHIDAYK 2: Deamidated (NQ)
1671.9	12166.27	-3.28	362 - 375	DQVILLNKHIDAYK 2: Deamidated (NQ) 7: Deamidated (NQ)
1684.89	60314.71	-2.75	142 - 157	DGIIWVATEGALNTPK
1687.87	12211.43	-2.68	224 - 240	MAGNGGDAALALLLLDR 1: Oxidation (M) 4: Deamidated (NQ)
1788.85	31278.36	-2.27	292 - 307	GPEQTQGNFGDQELIR
1823.82	6976	-0.94	29 - 46	ITFGGPSDSTGSNQNGER
1824.8	28961.85	-2.56	29 - 46	ITFGGPSDSTGSNQNGER 13: Deamidated (NQ)
1861.95	12083.4	-2.51	403 - 419	QQTVTLLPAADLDDFSK
1944.95	180056.74	-5.54	291 - 307	RGPEQTQGNFGDQELIR
1990.05	9214.58	-0.87	402 - 419	KQQTVTLLPAADLDDFSK

2015.08	53623.51	2.75	353 - 369	LDDKDPNFKDQVILLNK
2025.04	30090.62	0.06	334 - 352	IGMEVTPSGTWLTYTGAIK
2041.03	22198.44	-2.68	334 - 352	IGMEVTPSGTWLTYTGAIK 3: Oxidation (M)
2060.15	34543.96	-0.75	164 - 183	NPANNAAIVLQLPQGTTLPK
2181.02	34538.78	-4.81	83 - 102	GQGVPINTNSSPDDQIGYYR
2252.07	2553.02	6.62	314 - 333	HWPQIAQFAPSASAFFGMSR 18: Oxidation (M)
2267.08	10035.9	2.33	122 - 141	WYFYYLGTGPEAGLPYGANK
2304.01	1250	-1.06	420 - 439	QLQQSMSSADSTQAHHHHHH
2324.19	65313.93	5.77	55 - 75	RPQGLPNNTASWFTALTQHGK
2658.11	1057	-12.64	2 - 24	GSSHHHHHHSQDQMSDNGPQNQR

Table S4 - Detected ions (MH+) and matched peptides from the recombinant N protein (P.1) according to peptide mapping. The range of residues corresponding to the peptides, the relative intensity of the ion, the mass deviation (in parts per million, ppm), as well as possible peptide modifications (oxidation and deamidation) are indicated. Peptides that differ in at least one of the studied N proteins are marked in red, and unique peptides for this recombinant proteins are marked in bold red.

MH^+	Intensity (RU)	Mass deviation (ppm)	Range of residues	Sequence
531.29	4914.45	3.81	219 - 223	TSPAR
749.35	5600.24	0.23	192 - 199	GGSQASSR
886.41	68324.31	0.93	184 - 191	GFYAEGSR
904.49	11590.49	3.49	76 - 82	EDLKFPR
916.48	6706.34	4.58	376 - 383	TFPPTEPK
933.47	6341.02	2.08	210 - 218	NSTPGSSKR
**1029.46**	**5834.65**	**2.06**	**95 - 102**	**DDQIGYYR**
1126.56	145246.58	4.16	281 - 290	AYNVTQAFGR
1128.56	20454.76	10.83	390 - 399	ADETQALPQR
1185.56	9703.26	4.26	95 - 103	DDQIGYYRR
1202.61	5605.11	4.04	252 - 262	GQQQQGQTVTK
**1229.62**	**14521.15**	**5.99**	**83 - 94**	**GQGVPINTNSSR**
1256.66	21467.25	6.69	389 - 399	KADETQALPQR
1330.71	5722.49	4.46	252 - 263	GQQQQGQTVTKK
1384.75	13517.71	3.92	388 - 399	KKADETQALPQR
1527.79	2245.15	4.54	277 - 290	TATKAYNVTQAFGR
1605.8	2216.66	6.82	248 - 262	MSGKGQQQQGQTVTK
1616.74	4199.39	3.83	184 - 199	GFYAEGSRGGSQASSR
1643.84	3705.69	1.61	370 - 383	HIDAYKTFPPTEPK
1684.89	5885.92	1.25	142 - 157	DGIIWVATEGALNTPK
1788.85	10577.79	4.52	292 - 307	GPEQTQGNFGDQELIR
1823.82	36601	2.45	29 - 46	ITFGGPSDSTGSNQNGER
1824.8	81532	6.82	29 - 46	ITFGGPSDSTGSNQNGER 13: Deamidated (NQ)

1861.95	2471.9	1.07	403 - 419	QQTVTLLPAADLDDFSK
1944.95	55336.74	0.62	291 - 307	RGPEQTQGNFGDQELIR
1990.05	1560.49	-1.42	402 - 419	KQQTVTLLPAADLDDFSK
2015.08	1671	3.46	353 - 369	LDDKDPNFKDQVILLNK
2025.04	1452	-0.23	334 - 352	IGMEVTPSGTWLTYTGAIK
2060.15	7809.62	-1.11	164 - 183	NPANNAAIVLQLPQGTTLPK
2195.99	7738.64	-4.31	29 - 50	ITFGGPSDSTGSNQNGERSGAR 13: Deamidated (NQ)
**2240.07**	**6763.77**	**0.26**	**83 - 102**	**GQGVPINTNSSRDDQIGYYR**
2262.05	1649	0.84	25 - 46	NAPRITFGGPSDSTGSNQNGER
2263.03	2903.23	-1.03	25 - 46	NAPRITFGGPSDSTGSNQNGER 1: Deamidated (NQ)
2304.01	2247	-6.29	420 - 439	QLQQSMSSADSTQAHHHHHH
2324.19	7252.47	5.37	55 - 75	RPQGLPNNTASWFTALTQHGK
**2396.17**	**2686.4**	**-5.64**	**83 - 103**	**GQGVPINTNSSRDDQIGYYRR**
2658.11	13583.29	-6.24	2 - 24	GSSHHHHHHSQDQMSDNGPQNQR
2674.11	3517.95	-10.12	2 - 24	GSSHHHHHHSQDQMSDNGPQNQR 14: Oxidation (M)
2739.49	2364.49	4.96	158 - 183	DHIGTRNPANNAAIVLQLPQGTTLPK
2809.44	1317.17	-3.08	55 - 79	RPQGLPNNTASWFTALTQHGKEDLK
2927.54	839.91	-11.71	164 - 191	NPANNAAIVLQLPQGTTLPKGFYAEGSR

Table S5 - Detected ions (MH+) and matched peptides from the recombinant N protein (B.1.617.2) according to peptide mapping. The range of residues corresponding to the peptides, the relative intensity of the ion, the mass deviation (in parts per million, ppm), as well as possible peptide modifications (oxidation and deamidation) are indicated. Peptides that differ in at least one of the studied N proteins are marked in red, and unique peptides for this recombinant proteins are marked in bold red.

MH^+	Intensity (RU)	Mass deviation (ppm)	Range of residues	Sequence
587.32	135990.03	4.92	117 - 121	DLSPR
663.33	19335.88	3.89	264 - 270	SAAEASK
698.36	122556.76	1.98	158 - 163	DHIGTR
711.33	27967.01	2.6	308 - 313	QGTDYK
746.38	77014.8	1.9	370 - 375	HIDAYK
749.36	177458.24	2	192 - 199	GGSQASSR
831.46	108199.36	1.08	241 - 247	LNQLESK
**846.48**	**99291.25**	**0.1**	**76 - 82**	**EGLKFPR**
862.44	25509.04	-1.44	115 - 121	MKDLSPR 1: Oxidation (M)
886.41	756710.07	4.72	184 - 191	GFYAEGSR
916.48	287194.01	2.08	376 - 383	TFPPTEPK
942.56	85270.98	1.59	362 - 369	DQVILLNK
977.51	10530.36	-13.02	108 - 116	IRGGDGKMK 8: Oxidation (M)
1091.54	112672.57	-0.66	353 - 361	LDDKDPNFK
1126.57	715453.11	2.1	281 - 290	AYNVTQAFGR

**1148.58**	**226752.4**	**-9.83**	**390 - 399**	**AYETQALPQK**
1202.61	186911.59	-0.82	252 - 262	GQQQQGQTVTK
**1276.68**	**20324.61**	**-5.59**	**389 - 399**	**KAYETQALPQK**
**1349.61**	**50180.87**	**-2**	**210 - 223**	**NSTPGSSMGTSPAR**
**1365.6**	**97730.22**	**-4.01**	**210 - 223**	**NSTPGSSMGTSPAR 8: Oxidation (M)**
1671.87	79458.02	-3.37	224 - 240	MAGNGGDAALALLLLDR 4: Deamidated (NQ)
1684.88	266414.18	2.92	277 - 291	TATKAYNVTQAFGRR 7: Deamidated (NQ)
1687.86	255134.92	-4.31	224 - 240	MAGNGGDAALALLLLDR 1: Oxidation (M) 4: Deamidated (NQ)
1788.85	283129.9	-0.66	292 - 307	GPEQTQGNFGDQELIR
**1810.83**	**11259.8**	**15.18**	**206 - 223**	**NSSRNSTPGSSMGTSPAR 1: Deamidated (NQ) 12: Oxidation (M)**
1823.82	53543	0.15	29 - 46	ITFGGPSDSTGSNQNGER
1824.8	399973.72	-0.52	29 - 46	ITFGGPSDSTGSNQNGER 13: Deamidated (NQ)
1861.95	61168.59	-4.54	403 - 419	QQTVTLLPAADLDDFSK
1944.95	441339.68	-0.78	291 - 307	RGPEQTQGNFGDQELIR
1990.05	31220.51	-1.16	402 - 419	KQQTVTLLPAADLDDFSK
2015.08	66803.83	-1.51	353 - 369	LDDKDPNFKDQVILLNK
2025.03	12975.21	-1.71	334 - 352	IGMEVTPSGTWLTYTGAIK
2041.03	28249.29	-1.55	334 - 352	IGMEVTPSGTWLTYTGAIK 3: Oxidation (M)
2060.15	71424.99	-2.3	164 - 183	NPANNAAIVLQLPQGTTLPK
2181.03	323546.63	4.97	83 - 102	GQGVPINTNSSPDDQIGYYR
2658.15	1205	13.92	2 - 24	GSSHHHHHHSQDQMSDNGPQNQR
2674.13	2832	8.77	2 - 24	GSSHHHHHHSQDQMSDNGPQNQR 14: Oxidation (M)

Table S6 - Detected ions (MH+) and matched peptides from the recombinant N protein (B.1.1.529) according to peptide mapping. The range of residues corresponding to the peptides, the relative intensity of the ion, the mass deviation (in parts per million, ppm), as well as possible peptide modifications (oxidation and deamidation) are indicated. Peptides that differ in at least one of the studied N proteins are marked in red, and unique peptides for this recombinant proteins are marked in bold red.

MH^+	Intensity (RU)	Mass deviation (ppm)	Range of residues	Sequence
531.29	26371.43	-3.03	216 - 220	TSPAR
587.31	40219	-1.59	114 - 118	DLSPR
663.33	5896.69	-1.44	261 - 267	SAAEASK
698.36	38888.37	-2.08	155 - 160	DHIGTR
711.33	8824.8	-3.23	305 - 310	QGTDYK
746.38	21175.67	-2.63	367 - 372	HIDAYK
749.35	59926.69	-0.86	189 - 196	GGSQASSR
777.37	4846	-3.1	207 - 214	NSTPGSSK
831.45	31441.7	-2.71	238 - 244	LNQLESK
862.44	7317.23	-3.59	112 - 118	MKDLSPR 1: Oxidation (M)
886.41	363197.76	2.38	181 - 188	GFYAEGSR

904.49	101311.12	-0.54	73 - 79	EDLKFPR
916.48	112029.82	-1.69	373 - 380	TFPPTEPK
942.56	32096.42	-1.03	359 - 366	DQVILLNK
977.51	6207.8	-14.97	105 - 113	IRGGDGKMK 8: Oxidation (M)
1091.54	70287.24	-1.49	350 - 358	LDDKDPNFK
1126.57	363043.29	2.15	278 - 287	AYNVTQAFGR
1128.57	124535.85	5.55	387 - 396	ADETQALPQR
1202.61	56376.21	-2.56	249 - 259	GQQQQGQTVTK
1256.66	95743.6	1.25	386 - 396	KADETQALPQR
1607.8	7144.16	17.56	245 - 259	MSGKGQQQQGQTVTK 6: Deamidated (NQ) 7: Deamidated (NQ)
1671.87	5943.5	-2.39	221 - 237	MAGNGGDAALALLLLDR 4: Deamidated (NQ)
1684.88	18260.63	-3.3	139 - 154	DGIIWVATEGALNTPK
1687.87	55851.77	-0.67	221 - 237	MAGNGGDAALALLLLDR 1: Oxidation (M) 4: Deamidated (NQ)
1788.86	213813.42	2.74	289 - 304	GPEQTQGNFGDQELIR
**1824.81**	**5665.32**	**3.03**	**29 - 47**	**ITFGGPSDSTGSNQNGGAR 13: Deamidated (NQ) 14: Deamidated (NQ)**
1861.95	21329.44	-3.79	400 - 416	QQTVTLLPAADLDDFSK
1944.96	278052.48	3.29	288 - 304	RGPEQTQGNFGDQELIR
1990.05	9373.06	-0.08	399 - 416	KQQTVTLLPAADLDDFSK
2015.09	38544.41	2.13	350 - 366	LDDKDPNFKDQVILLNK
2025.04	14201.32	0.26	331 - 349	IGMEVTPSGTWLTYTGAIK
2041.03	38807.02	-0.83	331 - 349	IGMEVTPSGTWLTYTGAIK 3: Oxidation (M)
2060.15	32199.1	-0.24	161 - 180	NPANNAAIVLQLPQGTTLPK
2181.04	150735.97	9.55	80 - 99	GQGVPINTNSSPDDQIGYYR
2267.1	35118.93	7.33	119 - 138	WYFYYLGTGPEAGLPYGANK
2320.02	3520.32	8.72	417 - 436	QLQQSMSSADSTQAHHHHHH 6: Oxidation (M)
2325.21	5091.31	17.86	52 - 72	RPQGLPNNTASWFTALTQHGK 3: Deamidated (NQ)
2356.21	8503.4	-14.58	215 - 237	RTSPARMAGNGGDAALALLLLDR 7: Oxidation (M) 10: Deamidated (NQ)
2658.16	1069	18.2	2 - 24	GSSHHHHHHSQDQMSDNGPQNQR
2674.16	1927	19.57	2 - 24	GSSHHHHHHSQDQMSDNGPQNQR 14: Oxidation (M)

Table S7 - Detected ions (MH+) and matched peptides from the recombinant N protein (HCoV OC43) according to peptide mapping. The range of residues corresponding to the peptides, the relative intensity of the ion, the mass deviation (in parts per million, ppm), as well as possible peptide modifications (oxidation and deamidation) are indicated. Peptides that differ in at least one of the studied N proteins are marked in red, and unique peptides for this recombinant proteins are marked in bold red.

MH^+	Intensity (RU)	Mass deviation (ppm)	Range of residues	Matched peptide
564.26	4639.77	-28.41	22 - 26	QSSSR
573.35	4359.95	-25.83	343 - 347	LELAK

591.28	6763.99	-9.95	27 - 32	ASSGNR
599.35	6470.51	-26.62	56 - 60	RAQPK
616.34	5974.3	-6.71	432 - 436	VQQNK
617.34	32674.01	-2.34	49 - 53	NVQTR
626.4	73147.01	7.78	132 - 136	QLLPR
631.31	9566.58	-10.94	208 - 213	SAPNSR
635.3	6551.06	-13.28	222 - 228	ASSAGSR
689.38	5090.85	-3.54	33 - 39	SGNGILK 3: Deamidated (NQ)
693.37	6743	5.34	367 - 372	YNGAIR
694.37	27035	22.8	367 - 372	YNGAIR 2: Deamidated (NQ)
740.48	37929.29	7.79	280 - 285	ILNKPR
744.36	344721.45	19.17	113 - 117	GYWYR
761.37	31912.34	19.78	125 - 131	TADGNQR
794.42	16695.59	18.86	361 - 366	DVYELR
1104.52	417839.94	14.61	40 - 48	WADQSDQVR
1115.62	10261.13	12.64	261 - 270	DATKPQQVTK
1142.48	13506.72	-13.49	293 - 302	QCTVQQCFGK 1: Deamidated (NQ)
1187.56	6595.45	8.3	179 - 189	DPSSDEAIPTR
1231.7	15975.26	21	439 - 449	ELTAEDISLLK
1348.65	9673.61	14.84	304 - 316	GPNQNFGGGEMLK
1364.65	5895.44	17.36	304 - 316	GPNQNFGGGEMLK 11: Oxidation (M)
1425.71	3826.23	10.57	348 - 360	VQNLSGNPDEPQK
1475.75	24007.45	26.79	373 - 385	FDSTLSGFETIMK
1491.75	13844.14	32.7	373 - 385	FDSTLSGFETIMK 12: Oxidation (M)
1504.76	39343.8	15.37	303 - 316	RGPNQNFGGGEMLK
1520.76	20933.59	24.5	303 - 316	RGPNQNFGGGEMLK 12: Oxidation (M)
1542.77	7187.07	20.36	415 - 429	NGQGENDNISVAVPK 1: Deamidated (NQ)
1602.82	12880.19	27.65	137 - 149	WYFYYLGTGPHAK
1938.04	33934.73	-2.64	261 - 277	DATKPQQVTKHTAKEVR 6: Deamidated (NQ)
2115.17	1108.38	34.72	237 - 257	TPTSGVTPDMADQIASLVLAK
2201.13	6827.92	21.93	348 - 366	VQNLSGNPDEPQKDVYELR
2272.21	1618.75	27.64	91 - 112	EFEFVEGQGVPIAPGVPATEAK
2380.06	707	19.36	450 - 468	KMDEPYTEDTSEIHHHHHH
2457.3	8076.09	13.44	89 - 112	GKEFEFVEGQGVPIAPGVPATEAK
2710.4	3728.05	5.36	317 - 342	LGTSDPQFPILAELAPTAGAFFFGSR
2806.32	2412.79	4.64	386 - 409	VLNENLNAYQQQDGMMNMSPKPQR
2822.33	1805.77	10.24	386 - 409	VLNENLNAYQQQDGMMNMSPKPQR 15: Oxidation (M)
2838.35	2526.04	18.5	386 - 409	VLNENLNAYQQQDGMMNMSPKPQR 15: Oxidation (M) 16: Oxidation (M)
2854.34	2133.97	17.73	386 - 409	VLNENLNAYQQQDGMMNMSPKPQR 15: Oxidation (M) 16: Oxidation (M) 18: Oxidation (M)
3106.59	2833.05	24.96	179 - 207	DPSSDEAIPTRFPPGTVLPQGYYIEGSGR

3197.56	8097.49	30.52	150 - 178	DQYGTDIDGVYWVASNQADVNTPADIVDR

References

1. Kozlovskaya, L.; Piniaeva, A.; Ignatyev, G.; Selivanov, A.; Shishova, A.; Kovpak, A.; Gordeychuk, I.; Ivin, Y.; Berestovskaya, A.; Prokhortchouk, E.; et al. Isolation and phylogenetic analysis of SARS-CoV-2 variants collected in Russia during the COVID-19 outbreak. *International journal of infectious diseases : IJID : official publication of the International Society for Infectious Diseases* **2020**, *99*, 40-46, doi:10.1016/j.ijid.2020.07.024.
